# Cold-water immersion (cryotherapy) for preventing and treating muscle soreness after exercise

**DOI:** 10.1590/S1516-31802012000500015

**Published:** 2012-11-13

**Authors:** Chris Bleakley, Suzanne McDonough, Evie Gardner, David G. Baxter, Ty J. Hopkins, Gareth W. Davison

## Abstract

**BACKGROUND:**

Many strategies are in use with the intention of preventing or minimizing delayed onset muscle soreness and fatigue after exercise. Cold-water immersion, in water temperatures of less than 15 °C, is currently one of the most popular interventional strategies used after exercise.

**OBJECTIVES:**

To determine the effects of cold-water immersion in the management of muscle soreness after exercise.

**SEARCH METHODS:**

In February 2010, we searched the Cochrane Bone, Joint and Muscle Trauma Group Specialised Register, the Cochrane Central Register of Controlled Trials (The Cochrane Library (2010, Issue 1), Medline, Embase, Cumulative Index to Nursing and Allied Health (CINAHL), British Nursing Index and archive (BNI), and the Physiotherapy Evidence Database (PEDro). We also searched the reference lists of articles, handsearched journals and conference proceedings and contacted experts. In November 2011, we updated the searches of Central (2011, Issue 4), Medline (up to November Week 3 2011), Embase (to 2011 Week 46) and CINAHL (to 28 November 2011) to check for more recent publications.

**SELECTION CRITERIA:**

Randomized and quasi-randomized trials comparing the effect of using cold-water immersion after exercise with: passive intervention (rest/no intervention), contrast immersion, warm-water immersion, active recovery, compression, or a different duration/dosage of cold-water immersion. Primary outcomes were pain (muscle soreness) or tenderness (pain on palpation), and subjective recovery (return to previous activities without signs or symptoms).

**DATA COLLECTION AND ANALYSIS:**

Three authors independently evaluated study quality and extracted data. Some of the data were obtained following author correspondence or extracted from graphs in the trial reports. Where possible, data were pooled using the fixed-effect model.

**MAIN RESULTS:**

Seventeen small trials were included, involving a total of 366 participants. Study quality was low. The temperature, duration and frequency of cold-water immersion varied between the different trials as did the exercises and settings. The majority of studies failed to report active surveillance of pre-defined adverse events. Fourteen studies compared cold-water immersion with passive intervention. Pooled results for muscle soreness showed statistically significant effects in favour of cold-water immersion after exercise at 24 hour (standardized mean difference, SMD -0.55, 95% CI -0.84 to -0.27; 10 trials), 48 hour (SMD -0.66, 95% CI -0.97 to -0.35; 8 trials), 72 hour (SMD -0.93; 95% CI -1.36 to -0.51; 4 trials) and 96 hour (SMD -0.58; 95% CI -1.00 to -0.16; 5 trials) follow-ups. These results were heterogeneous. Exploratory subgroup analyses showed that studies using cross-over designs or running-based exercises showed significantly larger effects in favour of coldwater immersion. Pooled results from two studies found cold-water immersion groups had significantly lower ratings of fatigue (MD - 1.70; 95% CI -2.49 to -0.90; 10 units scale, best to worst), and potentially improved ratings of physical recovery (MD 0.97; 95% CI -0.10 to 2.05; 10 units scale, worst to best) immediately after the end of cold-water immersion. Five studies compared cold-water with contrast immersion. Pooled data for pain showed no evidence of differences between the two groups at four follow-up times (immediately, 24, 48 and 72 hours after treatment). Similar findings for pooled analyses at 24, 48 and 72 hour follow-ups applied to the four studies comparing cold-water with warm-water immersion. Single trials only compared coldwater immersion with respectively active recovery, compression and a second dose of cold water immersion at 24 hours.

**AUTHORS’ CONCLUSIONS:**

There was some evidence that cold-water immersion reduces delayed onset muscle soreness after exercise compared with passive interventions involving rest or no intervention. There was insufficient evidence to conclude on other outcomes or for other comparisons. The majority of trials did not undertake active surveillance of pre-defined adverse events. High quality, well reported research in this area is required.

**This is the abstract of a Cochrane Review published in the Cochrane Database of Systematic Reviews (CDSR) 2012, issue 2, DOI:** 10.1002/14651858.CD008262.pub2. (http://onlinelibrary.wiley.com/doi/10.1002/14651858.CD008262.pub2/abstract). For full citation and authors’ details see reference 1.

**The full text is freely available from:** http://www.cochranejournalclub.com/cryotherapy-preventing-treating-muscle-soreness-exercise/pdf/CD008262.pdf
